# Literature for the Million

**Published:** 1895-05-18

**Authors:** 


### Literature for the Million.

We have free education; what have we done with it? We have insisted that every boy and girl shall learn to read, but what have we done to satisfy the appetite for reading thus created? In one way, very much. Among the silent revolutions which we profit by but never note is the great revolution in school books. Formerly school books were manufactured by the grubbiest of Grub Street hacks. They, indeed, contained information, but couched in so dull a form, so lacking in philosophy and proportion, that the hardest efforts of both teacher and pupils was necessary to assimilate it. Only of late years has it occurred to the best publishers to secure the best authors to provide for that immense public which occupies the benches of our national schools.

But, after all, school books are school books, associated with memories not wholly pleasant, and when they are once read the intellect craves for something new. What is offered it? The newspaper and the penny novelette. Other books there are, as the publishers' lists do show, but—even allowing that all of these are worth reading—how few are within the reach of that public we speak of, those who have received free education at a national school. Our milliners and 'prentices, however intellectual in their tastes, cannot afford to buy the masterpieces of literature, and, if they read at all, are compelled to read that which is at best ephemeral, and often worthless. We have now our free libraries in most towns, but what of the country districts where, more than in any city, books are needed to keep the brain from stagnating? Parsons will tell you that, in spite of our educational system, couples occasionally come to be married who cannot sign their names. Even those who do not fall to such utter ignorance become sluggish, narrow, and even brutal for sheer lack of imagination, and that wider vision of men and things which books alone can bring.

Therefore we welcome the plan suggested by Mr. Stead, in the *Review of Reviews*, of sending circulating library boxes to any place that will subscribe £6 per annum. The boxes will contain from forty to fifty volumes, and will be changed once a quarter. Some standard novels will be among the books, but the boxes, unlike Mudie's, will not be filled up with three-volume fiction. With the stories will be mingled history, travel, biography, and economics. Catholicity will be the keynote of the collection. It will range, in the words of the hackneyed quotation, "from grave to gay, from lively to severe," and will enable its readers to look at the world through the eyes of many men. The subscribing centre—Parish Council, School Board, church, employer, bookseller, group of friends, or single individual—can thus command 200 books a year for perusal and circulation, on condition, however, of charging not more than twopence a week for the loan of books, for Mr. Stead's intention is to supply not the well-to-do but the poor with reading matter. Doubtless, however, there are many not reckoned among those conventionally styled the working classes to whom this chance of seeing books of value will be no slight boon. Which of us is rich enough to buy all the books we want! And, indeed, only the rash man or the millionaire buys books from the recommendation of the catalogue; we read many books, only a few of which we ultimately desire to possess, and the library box would be the means of putting us on the track of what we really want. Besides ordinary books, it has occurred to Mr. Stead, and the idea is admirable, to include in his collection the bound volumes of our good magazines, which are a library in themselves. Twelve boxes of books are already prepared, so that a library of about 600 volumes is at the command of any centre that is prepared to pay £6 per annum. It has been pointed out that in country districts books cannot be exchanged quickly, and that therefore a new box would not be needed every quarter. If, then, a centre wishes to exchange books only twice a year it will save a pound, for as the subscription includes the box and the carriage of it to and from the centre, Mr. Stead deducts ten shillings for each extra quarter that the box is kept. For districts where even £5 or £6 seems too large a sum, and where, perhaps, a simpler kind of literature is wanted, Mr. Stead has prepared boxes which can be had and kept for six months on payment of £3 a year. Thus, like a wise man, he caters for all tastes and all purses.

Think what it means. As Mr. Stead says in his advertisement, "any group of thirty persons can, by paying a penny a week each, or a shilling a quarter, obtain on loan every quarter a fresh supply of forty-five of the best books in the world, costing £10." Ten pounds' worth of thought, fact, and fancy, for four shillings! Though we may be far from rich, there are few of us who cannot spare four shillings a year for anything we greatly wish. After this let no one complain that his poverty or his remoteness from the centres of the world's life prevents his reading good books.

But, besides the books which most people wish to read once, but few, except students of special subjects, require to have by them, there are others, the possession of which seems to bookish folk as necessary as the possession of a certain modicum of daily bread; books which not to know, and to know well, argues yourself illiterate. And many of these book-lovers—more loyal lovers than the throng who squander their affections and their cash on bindings and first editions—are poor. Mr. Stead, who has known the keen book-hunger himself, has a plan for these. It is the publication of what he calls the "Master-piece Library," and will begin with the "Penny Poets," with Macaulay, Scott, Byron, and Lowell; but we may safely hope to see some of our great prose-writers added to the list. The volumes will not contain complete editions of any of our authors, but Mr. Stead assures us that they will contain their best. Perhaps it is sad to think that the best of all but the greatest can be condensed into a penny volume; yet how many of us scribblers would rejoice to know that we could write even a pennyworth which those yet unborn would care to read! We would venture to offer a suggestion to Mr. Stead, namely, that he should include in his penny library translations from some of the great foreign writers. Many of us are Shakespearean in that we have "small Latin and less Greek"; yet we could enjoy the story of Dido and that of Nausicaa, we could appreciate Sophocles the divine and Euripides the human, as well as our own Shakespeare; we could laugh with Aristophanes and Molière, we could try to gauge the philosophy of Goethe, and the bitter wit of Heine. There is little doubt that the scheme will succeed, and that it will gladden many a dreary hearth, and lighten many a dark evening. It will give healthful recreation to many who now have to seek the *Police News* for information, and the public-house for society. It is to be expected that a good many superior people will mock at the scheme. That is natural. "What, in the name of the Bodleian, has the general public to do with literature?" asks Mr. Augustine Birrell. If you keep literature shut up within the Bodleian, certainly nothing; but if you send it into the highways and hedges, who knows how much!

